# Beyond Surveillance Mutations: Long-Term Trends and Clinical Relevance of Transmitted HIV Drug Resistance in Spain (2007–2023)

**DOI:** 10.1093/ofid/ofag296

**Published:** 2026-05-18

**Authors:** Paloma Muñoz Báez, Marta Illescas López, Adolfo de Salazar, Iker Falces Romero, Adriana Pinto, Irene Portilla, Mar Masiá, Joaquim Peraire, María Remedios Alemán Valls, Marta Sanchiz, Asunción Iborra, Begoña Baza, Antonio Aguilera, Julián Olalla, Nuria Espinosa, José Antonio Iribarren, Laura Gisbert, Arkaitz Imaz, Marta Montero, María Navarro Marcotegui, Inés Suárez-García, Melchor Riera, María Jesús Pérez Elias, Juan Carlos Galán, Lucio Jesús García Fraile, José Sanz, Sofía Ibarra Ugarte, Enrique Bernal, José Ramón Blanco, Gemma Navarro, Onofre Martínez, María Isabel Viciana Ramos, Mohamed Omar Mohamen-Balghata, Elena Delgado, Michael M Thompson, Cristina Moreno, Cohorte CoRIS, Ana Fuentes, Federico García

**Affiliations:** Departamento de Microbiología Clínica, Hospital Universitario Clínico San Cecilio, Granada, Spain; Instituto de Investigación Ibs, Granada, Spain; Ciber de Enfermedades Infecciosas, CIBERINFEC, ISCIII, Madrid, Spain; Departamento de Microbiología Clínica, Hospital Universitario Clínico San Cecilio, Granada, Spain; Instituto de Investigación Ibs, Granada, Spain; Ciber de Enfermedades Infecciosas, CIBERINFEC, ISCIII, Madrid, Spain; Departamento de Microbiología Clínica, Hospital Universitario Clínico San Cecilio, Granada, Spain; Instituto de Investigación Ibs, Granada, Spain; Ciber de Enfermedades Infecciosas, CIBERINFEC, ISCIII, Madrid, Spain; Ciber de Enfermedades Infecciosas, CIBERINFEC, ISCIII, Madrid, Spain; Servivio de Microbiología Clínica, Hospital Universitario La Paz, Madrid, Spain; Unidad de Enfermedades Infecciosas, Hospital 12 de Octubre, Madrid, Spain; Unidad de Enfermedades Infecciosas, Hospital General Universitario de Alicante, Alicante, Spain; Ciber de Enfermedades Infecciosas, CIBERINFEC, ISCIII, Madrid, Spain; Unidad de Enfermedades Infecciosas, Hospital General Universitario de Elche, Elche, Spain; Ciber de Enfermedades Infecciosas, CIBERINFEC, ISCIII, Madrid, Spain; Unidad de Enfermedades Infecciosas, Hospital Universitari de Tarragona Joan XXIII, IISPV, Universitat Rovira i Virgili, Tarragona, Spain; Unidad de Enfermedades Infecciosas, Hospital Universitario de Canarias, San Cristobal de la Laguna, Spain; Unidad de Enfermedades Infecciosas, Hospital Universitari Vall d'Hebron, Barcelona, Spain; Departamento de Microbiología Clínica, Hospital Virgen de la Arrixaca, Murcia, Spain; Centro Sanitario Sandoval, Hospital San Carlos, Madrid, Spain; Departamento de Microbiología Clínica, Complejo Hospitalario Universitario de Santiago, Santiago, Spain; Unidad de Enfermedades Infecciosas, Hospital Costa del Sol, Marbella, Spain; Unidad de Enfermedades Infecciosas, Microbiología Clínica y Medicina Preventiva, Instituto de Biomedicina de Sevilla/Hospital Universitario Virgen del Rocío/CSIC/Universidad de Sevilla, Sevilla, Spain; Unidad de Enfermedades Infecciosas, Hospital Universitario Donostia, San Sebastián, Spain; Unidad de Enfermedades Infecciosas, Hospital Universitari Mutua Terrassa, Terrassa, Spain; Unidad de Enfermedades Infecciosas, Hospital Universitario de Bellvitge, Barcelona, Spain; Unidad de Enfermedades Infecciosas, Hospital Universitario La Fe, Valencia, Spain; Unidad de Enfermedades Infecciosas, Hospital Universitario de Navarra, Pamplona, Spain; Ciber de Enfermedades Infecciosas, CIBERINFEC, ISCIII, Madrid, Spain; Grupo de Enfermedades Infecciosas, Hospital Infanta Sofía, FIIB HUIS HUHEN, Departamento de Medicina, Facultad de Medicina, Salud y Deporte, Universidad Europea de Madrid, Madrid, Spain; Ciber de Enfermedades Infecciosas, CIBERINFEC, ISCIII, Madrid, Spain; Unidad de Enfermedades Infecciosas, Hospital Son Espases, Mallorca, Spain; Ciber de Enfermedades Infecciosas, CIBERINFEC, ISCIII, Madrid, Spain; Unidad de Enfermedades Infecciosas, Hospital Ramón y Cajal, Madrid, Spain; Unidad de Enfermedades Infecciosas, Hospital Ramón y Cajal, Madrid, Spain; Ciber de Epidemiologia y Salud Publica, CIBERESP, Madrid, Spain; Ciber de Enfermedades Infecciosas, CIBERINFEC, ISCIII, Madrid, Spain; Unidad de Enfermedades Infecciosas, Hospital La Princesa, Madrid, Spain; Unidad de Enfermedades Infecciosas, Hospital Universitario Príncipe de Asturias, Madrid, Spain; Unidad de Enfermedades Infecciosas, Hospital Universitario Basurto, Bilbao, Spain; Unidad de Medicina Interna, Hospital Reina Sofía, Murcia, Spain; Unidad de Enfermedades Infecciosas, Hospital San Pedro Centro de Investigación Biomédica de La Rioja (CIBIR), Logroño, Spain; Área de Epidemiología y Enfermedades Crónicas, Hospital Universitari Parc Taulí, Sabadell, Spain; Unidad de Enfermedades Infecciosas, Hospital Universitario Santa Lucía, Cartagena, Spain; Unidad de Enfermedades Infecciosas, Hospital Universitario Virgen de la Victoria, Málaga, Spain; Unidad de Enfermedades Infecciosas, Complejo Hospitalario de Jaén, Jaén, Spain; Centro Nacional de Microbiologia, Instituto de Salud Carlos III, Madrid, Spain; Centro Nacional de Microbiologia, Instituto de Salud Carlos III, Madrid, Spain; Ciber de Enfermedades Infecciosas, CIBERINFEC, ISCIII, Madrid, Spain; Centro Nacional de Microbiologia, Instituto de Salud Carlos III, Madrid, Spain; Centro Coordinador CoRIS, Instituto de Salud Carlos III, Madrid, Spain; Departamento de Microbiología Clínica, Hospital Universitario Clínico San Cecilio, Granada, Spain; Instituto de Investigación Ibs, Granada, Spain; Ciber de Enfermedades Infecciosas, CIBERINFEC, ISCIII, Madrid, Spain; Departamento de Microbiología Clínica, Hospital Universitario Clínico San Cecilio, Granada, Spain; Instituto de Investigación Ibs, Granada, Spain; Ciber de Enfermedades Infecciosas, CIBERINFEC, ISCIII, Madrid, Spain

**Keywords:** HIV, integrase, PrEP, resistance, Test & Treat

## Abstract

**Background:**

While surveillance drug resistance mutations (SDRMs) remain essential for monitoring transmitted drug resistance (TDR), the clinical impact of transmitted resistance for current first-line regimens has become increasingly important. We aimed to update estimates of TDR in Spain and assess its clinical impact over time.

**Methods:**

This is a nationwide observational study within the CoRIS cohort. We estimated the prevalence of SDRMs and clinically meaningful resistance (CMR) among antiretroviral therapy (ART)–naïve individuals with baseline genotypic resistance testing in 2022–2023 and evaluated annual trends in CMR to first-line regimens from 2007 to 2023. Clinically meaningful resistance was defined as Stanford HIVDB resistance level ≥ 3.

**Results:**

In 2022–2023, 1028 individuals were included; 82.8% were male, 63.2% MSM, and 72.2% subtype B. Surveillance drug resistance mutation prevalence was 14.4% (95% CI 12.2%–16.9%), driven mainly by NNRTI-SDRMs, while INSTI-SDRMs remained rare [0.5% (95% CI 0.1%–1.5%)]; CMR to recommended first-line regimens was very low. Over 2007–2023, CMR declined markedly, coinciding with adoption of second-generation integrase inhibitors. Rilpivirine-associated resistance mutations were detected in 7.0%, largely due to E138A (4.8%). M184V was detected in 0.8%; 6 of 8 cases occurred in persons with current or prior oral pre-exposure prophylaxis (PrEP), although numbers were small.

**Conclusions:**

In Spain, TDR remains stable, but its clinical impact on first-line regimens has markedly declined. These findings support Test & Treat strategies and immediate ART initiation with contemporary triple- or dual-drug regimens, while highlighting the need for targeted resistance assessment for rilpivirine-containing regimens and further research on prior PrEP exposure and selected NRTI resistance patterns.

Antiretroviral therapy (ART) guidelines, including those of the US Department of Health and Human Services (DHHS) [1], the European AIDS Clinical Society (EACS) [2], IAS–USA [3], the World Health Organization (WHO) [4], and the Spanish GeSIDA/SEIMC guidelines [5], recommend baseline HIV-1 drug resistance testing prior to initiating ART in all individuals. In general, this recommendation applies to resistance against reverse transcriptase inhibitors (RT) and protease inhibitors (PI), whereas integrase resistance testing is typically reserved for cases in which transmission from an index patient failing an integrase strand transfer inhibitor (INSTI)–based regimen is suspected. In Spain, however, many centers have progressively incorporated next-generation sequencing (NGS)–based resistance testing into routine baseline genotyping. Within these laboratory workflows, extending sequencing from RT and protease to include integrase entails little or no meaningful additional cost, which has favored broader availability of baseline INSTI resistance data beyond the specific scenarios contemplated in most guidelines. This broader testing strategy may provide useful information not only for individual patient management in selected cases but also for surveillance of transmitted INSTI resistance in the context of widespread INSTI use. More recently, baseline resistance testing has also been recommended in newly diagnosed individuals who have failed long-acting cabotegravir pre-exposure prophylaxis (PrEP) [1]. These recommendations are supported by evidence that identifying transmitted drug resistance (TDR) before ART initiation reduces the risk of treatment failure and is cost-effective [6].

Surveillance of TDR has historically relied on the surveillance drug resistance mutation (SDRM) list for reverse transcriptase and protease, established in 2007 and updated in 2009 by Bennett et al [7]. However, the introduction of INSTIs beginning with the approval of raltegravir in 2007 and its widespread adoption from 2009 onward fundamentally changed first-line ART composition [8]. Consequently, new frameworks and curated mutation lists have been proposed to monitor transmitted resistance affecting INSTIs [9].

The antiretroviral armamentarium has also expanded to include next-generation NNRTIs such as etravirine, rilpivirine, and doravirine, which exhibit distinct resistance-associated mutation (RAM) profiles not fully captured by the 2009 SDRM list [10]. In addition, after the introduction of long-acting injectable cabotegravir plus rilpivirine, first approved for clinical use in 2020–2021 as a switch strategy for virologically suppressed people with HIV-1 (PWH) [11–13], there has been increasing interest in rilpivirine-associated resistance mutations. These RAMs may persist as archived variants despite long-term viral suppression and have been associated with an increased risk of virological failure on cabotegravir/rilpivirine long-acting therapy. Consequently, current clinical guidelines emphasize the need to carefully evaluate both rilpivirine-associated RAMs and viral subtype when selecting candidates for long-acting cabotegravir/rilpivirine therapy [3, 14].

In addition, failures of oral PrEP with tenofovir plus lamivudine or emtricitabine have been associated with the selection of NRTI resistance mutations, particularly K65R and, most notably, M184V [15, 16]. Therefore, determining whether newly diagnosed individuals carrying these mutations have a history of PrEP failure is of special clinical interest.

In Spain, the CoRIS (Spanish HIV-1 Research Network Cohort) has performed annual surveillance of TDR in newly diagnosed, ART-naïve individuals since 2007, providing high-quality clinical, demographic, and virological information that enables detailed mapping of TDR patterns in the country [17–20]. In the present study, we update previously published estimates of transmitted resistance to NRTIs, NNRTIs, PIs, and INSTIs in Spain from 2007 onward, incorporating new data from the 2022–2023 period. This update extends previous national analyses through 2023. We also specifically analyze rilpivirine-associated resistance mutations and explore the relationship between the presence of M184V and prior exposure to oral PrEP. We further assess the predicted activity of currently recommended first-line ART regimens in Spain to support clinical decision-making and refine national guideline recommendations.

## METHODS

This multicenter, observational study was conducted within the framework of the Spanish HIV Research Network Cohort (CoRIS), an open, prospective cohort enrolling antiretroviral-naïve PWH aged over 13 and followed in HIV units across Spain. A detailed description of the cohort has been published previously [21]. For the 2022–2023 update analysis, we included ART-naïve individuals with available baseline HIV genotypic resistance data. Among 1225 ART-naïve individuals enrolled during this period, 197 (16.1%) were excluded because baseline genotypic resistance data were not available. All participants provided written informed consent, and the study received approval from the ethics committees of each participating center.

Participating centers provided HIV-1 sequence files in FASTA format, a standard text-based format used to represent nucleotide sequences, for protease (PR) and reverse transcriptase (RT), and integrase (IN), when available. All sequences derived from standard clinical genotyping assays and met medium or high-quality criteria. Sequence alignment, quality control, subtype assignment, and resistance interpretation were performed using the Stanford HIV-1 Drug Resistance Database (HIVDB; version 9.8.1; https://hivdb.stanford.edu/hivdb/by-sequences/). The prevalence of SDRMs was assessed using the Calibrated Population Resistance tool, applying the WHO-endorsed 2009 SDRM list. In addition, rilpivirine-associated RAMs were specifically assessed according to the mutation list proposed by the IAS–USA resistance guidelines [22]. Clinically relevant resistance to first-line ARVs was defined based on a Stanford algorithm resistance level of ≥3, including low-level resistance (level 3), intermediate resistance (level 4), and high-level resistance (level 5). Within the Stanford HIVDB framework, level 3 marks the onset of resistance, whereas levels 1 and 2 remain within the susceptible range in simplified classifications. This definition was intended to capture mutation patterns with plausible consequences for antiretroviral activity and regimen selection, although not necessarily virological failure, particularly in regimens with a high genetic barrier to resistance. Subtype assignment was conducted using the rapid HIVDB subtype tool, and ambiguous cases were resolved through phylogenetic reconstruction using a maximum likelihood–based phylogenetic tree, applying the Neighbor-Joining algorithm and the Jukes and Cantor substitution model with the CLC Genomics Workbench tool.

Clinical and demographic variables at enrollment were obtained from the CoRIS database, including gender, age, country of origin, educational level, HIV-1 transmission route, baseline CD4+ T-cell count, plasma HIV-1 RNA level, and HBV or HCV coinfection status. These variables were used to characterize the study population and to explore differences in the prevalence of SDRMs or clinically meaningful transmitted resistance using univariate analysis.

The primary outcome of interest was the prevalence of TDR, defined by the presence of SDRMs in any of the RT, PR, or IN sequences. Secondary outcomes included clinically meaningful resistance, considering only antiretroviral drugs included in first-line regimens recommended in Spain during each study period, and interpreting their predicted activity according to the Stanford HIVDB scoring system, the distribution of HIV-1 subtypes, the prevalence of rilpivirine-associated resistance mutations, and the assessment of the relationship between the presence of M184V mutation and prior exposure to oral PrEP. PrEP exposure is not systematically recorded in the CoRIS database; therefore, this information was specifically requested from recruiting centers only for individuals in whom the M184V mutation was detected. Prevalence estimates were accompanied by 95% confidence intervals, calculated using analytically derived variance estimators consistent with prior CoRIS methodological approaches.

Artificial intelligence (AI)–based tools were used exclusively for language editing and clarity improvement; no AI tools were used for data analysis, data interpretation, or decision-making.

## RESULTS

### Study Population and Prevalence of Transmitted Drug Resistance in 2022–2023

Between 2022 and 2023, a total of 1028 ART-naïve PWH were included in the demographic and virological analysis. Most individuals were male (82.8%), and men who have sex with men (MSM) represented the predominant transmission category (63.2%). The most frequent age group was 26–35 years (36.4%), and 41% of participants were of Spanish origin. Regarding immunovirological status at cohort entry, 44.5% PWH had CD4+ T-cell counts below 350 cells/mm^3^, while 45.2% had a plasma viral load > 100 000 copies/mL. The majority of individuals were infected with subtype B (72.2%). Among non-B subtypes, CRF02_AG was the most prevalent (6.3%), followed by subtype C (3.2%), subtype A1 (1.8%), A6 (1.7%), and subtype F (1.5%). Coinfection with hepatitis B virus (HBV) remained low (1.3%), and hepatitis C virus (HCV) coinfection was also infrequent (2.3%). Unless otherwise specified, percentages are calculated using the number of individuals with available data for each specific analysis; exact denominators are provided in the corresponding tables.

During the update study period, 456 RT, 457 PR, and 241 IN sequences were obtained in 2022 and 514 RT, 517 PR, and 335 IN sequences in 2023. Overall, baseline integrase sequences were available for 576 of 1028 participants (56.0%), with coverage increasing from 2022 to 2023, consistent with the progressive incorporation of broader baseline NGS-based resistance testing in participating centers. The overall prevalence of TDR during 2022–2023 was 14.4% (95% CI 12.2%–16.9%). Class-specific SDRM prevalence was 4.8% (95% CI 3.5%–6.4%) for NRTIs, 8.6% (95% CI 6.8%–10.6%) for NNRTIs, 1.5% (95% CI .9%–2.5%) for PIs, and 0.5% (95% CI .1%–1.5%) for INSTIs. Detailed SDRM distributions are summarized in Table 1. For NRTIs, the most frequently detected SDRMs included M41L [1.3% (95% CI .7%–2.3%)], T215 revertants D/E/I/C [1.2% (95% CI .6%–2.1)], and M184V [0.8% (95% CI .4%–1.6%)]. NNRTI-associated SDRMs were dominated by K103N/S [6.3% (95% CI 4.8–8.0], followed by G190A/E [1.1% (95% CI .6%–2.0%], and K101E [0.3% (95% CI .1%–.9%]. PI-associated SDRMs were infrequent, with D30N, I85V, I54V, M46I/L, F53L, V82A, and L90M observed at low prevalence (≤0.5%). For INSTIs, a single individual carried E138T/G140S/Q148H [0.2% (95% CI .0%–1.0%)].

**Table 1.** Prevalence of Surveillance Drug Resistance Mutations (SDRMs) Detected in ART-Naïve People With HIV-1 Included in CoRIS During 2022–2023, Stratified by Antiretroviral Drug Class

NRTI (n = 970)	n	% (95% CI)	NNRTI (n = 970)	n	% (95% CI)
D67N	2	0.2 (0.0–0.7)	G190A	10	1 (0.5–1.9)
L210W	4	0.4 (0.1–1.1)	G190E	1	0.1 (0.0–0.6)
M184V	8	0.8 (0.4–1.6)	K101E	3	0.3 (0.1–0.9)
M41L	13	1.3 (0.7–2.3)	K103N	53	5.5 (4–7.1)
T215 D/E/I/C	12	1.2 (0.6–2.1)	K103S	8	0.8 (0.4–1.6)
T215Y	2	0.2 (0.0–0.7)	Y181C	2	0.2 (0.0–0.7)
K219Q	1	0.1 (0.0–0.6)	Y188L	1	0.1 (0.0–0.6)
L74V	1	0.1 (0.0–0.6)	L100I	1	0.1 (0.0–0.6)
K219E	1	0.1 (0.0–0.6)	V106A	1	0.1 (0.0–0.6)
K65R	1	0.1 (0.0–0.6)	V106M	1	0.1 (0.0–0.6)
K70R	1	0.1 (0.0–0.6)	P225H	2	0.2 (0.0–0.7)
F77L	1	0.1 (0.0–0.6)	>1 SDRM	9	0.9 (0.4–1.8)
>1 SDRM	15	1.5 (0.9–2.6)	Total	83	8.6 (6.8–10.6)
Total	47	4.8 (3.5–6.4)			

IN (n = 576)	n	% (CI 95%)	RPV RAMs (n = 970)	n	% (CI 95%)
E138T	1	0.2 (0.0–1.0)	K101E	3	0.3 (0.0–0.9)
G140S	1	0.2 (0.0–1.0)	K101R	2	0.2 (0.0–0.7)
Q148H	1	0.2 (0.0–1.0)	K101P	5	0.5 (0.2–1.2)
>1 SDRM	1	0.2 (0.0–1.0)	E138A	47	4.8 (3.6–6.4)
Total	3	0.5 (0.1–1.5)	E138EK	1	0.1 (0.0–0.6)
PI (n = 974)	n	% (CI 95%)	E138EG	1	0.1 (0.0–0.6)
D30N	2	0.2 (0.0–0.7)	Y181C	2	0.2 (0.0–0.7)
I85V	2	0.2 (0.0–0.7)	H221HY	3	0.3 (0.0–0.9)
I54V	2	0.2 (0.0–0.7)	L100I	1	0.1 (0.0–0.6)
M46I	2	0.2 (0.0–0.7)	E138G	1	0.1 (0.0–0.6)
M46L	1	0.1 (0.0–0.6)	Y188L	1	0.1 (0.0–0.6)
F53L	5	0.5 (0.2–1.2)	Total	68	7.0 (5.4–8.9)
L90M	1	0.1 (0.0–0.6)			
>1 SDRM	1	0.1 (0.0–0.5)			
Total	15	1.5 (0.9–2.5)			

Data are presented as *n* (%) with corresponding 95% confidence intervals (95% CI). For NNRTIs SDRMs, mutations highlighted in bold correspond to rilpivirine-associated resistance mutations (RAMs) as defined by the International Antiviral Society–USA (IAS–USA) guidelines, including mutations not captured by the 2009 Bennett SDRM list. RAMs to rilpivirine, as defined by the IAS list are also shown.

Abbreviations: INSTI, integrase strand transfer inhibitor; NNRTI, non-nucleoside reverse transcriptase inhibitor; NRTI, nucleoside/nucleotide reverse transcriptase inhibitor; PI, protease inhibitor; SDRM, surveillance drug resistance mutation.

### Rilpivirine-Associated Mutations, M184V, and Clinically Meaningful Resistance

Rilpivirine-associated resistance mutations (RAMs), as defined by the IAS–USA guidelines, were detected in 7.0% (95% CI 5.4%–8.9%) of individuals, with E138A being the most prevalent 4.8 (95% CI 3.6–6.4). In addition, several rilpivirine RAMs not included in the Bennett et al 2009 SDRM list [7] were identified, including substitutions K101E/P, E138G/K/Q/R, Y181C, and H221Y.

M184V mutation was identified in 8 individuals [0.8% (95% CI: 0.4%–1.6%)], and 6 were current or former users of PrEP. When the data were analyzed by study year, 2 individuals carried M184V in 2022 [0.4% (95% CI: 0.0%–1.6%)], one of whom had current or prior PrEP use. In contrast, in 2023, 6 individuals were identified with the M184V mutation [1.2% (95% CI: 0.4%–2.5%)]; among these, only one individual had no history of PrEP use. Given the small absolute number of M184V cases, these findings should be interpreted cautiously. In addition, detailed PrEP exposure data were specifically reviewed for individuals with M184V and were not systematically collected for the remainder of the cohort; therefore, these observations are descriptive and should be considered hypothesis-generating rather than definitive evidence of an association at the population level.

The prevalence of clinically meaningful transmitted resistance (Stanford level ≥ 3) is presented in Table 2. For NRTIs, clinically meaningful transmitted resistance was 1.0% (95% CI .6%–2.0%). NNRTIs showed the highest burden of clinically meaningful transmitted resistance [8.0% (95% CI 6.4%–10.0%)]. For INSTIs, resistance was observed at 0.2% (95% CI .0%–1.0%) for dolutegravir and bictegravir, 0.4% (95%IC .0%–1.3%) for cabotegravir, and 1.7% (95% CI .8%–3.2%) for raltegravir. PI resistance remained extremely low at 0.1% (95% CI .0%–.6%). Detailed drug-specific estimates are shown in Table 2.

**Table 2.** Prevalence of Clinically Relevant Transmitted Resistance to Antiretroviral Drugs Recommended for First-Line Therapy in Spain Among ART-Naïve People With HIV-1 Included in CoRIS During 2022–2023

INSTI (n = 576)	n	% (CI 95%)
Raltegravir	10	1.7 (0.8–3.2)
Dolutegravir	1	0.2 (0.0–1.0)
Bictegravir	1	0.2 (0.0–1.0)
Cabotegravir	2	0.4 (0.0–1.3)
Total	10	1.7 (0.8–3.2)

NRTI (n = 970)	n	95% CI
Tenofovir alafenamide	2	0.2 (0.0–0.7)
Abacavir	8	0.8 (0.4–1.6)
Lamivudine/emtricitabine	8	0.8 (0.4–1.6)
Total	10	1.0 (0.6–2.0)

NNRTI (n = 970)	n	95% CI
Efavirenz	78	8.0 (6.4–10.0)
Rilpivirine	64	6.6 (5.1–8.4)
Doravirine	13	1.3 (0.7–2.3)
Total	78	8.0 (6.4–10.0)

PI (n = 974)	n	95% CI
Lopinavir	1	0.1 (0.0–0.6)
Atazanavir	1	0.1 (0.0–0.6)
Total	1	0.1 (0.0–0.6)

CMR was defined as a Stanford HIV-1 Drug Resistance Database level ≥ 3 (v9.8.1). Data are presented as *n* (%) with corresponding 95% confidence intervals (95% CI) and are shown by individual antiretroviral drug and drug class.

Abbreviations: INSTI, integrase strand transfer inhibitor; NNRTI, non-nucleoside reverse transcriptase inhibitor; NRTI, nucleoside/nucleotide reverse transcriptase inhibitor; PI, protease inhibitor.

### Univariate Comparisons and Temporal Trends in Clinically Meaningful Resistance

Differences in the prevalence between demographic, clinical, and virological variables and the presence of SDRMs or clinically meaningful transmitted resistance were assessed using univariate analyses (Table 3). Significant univariate differences were observed according to selected demographic, clinical, and virological characteristics, including transmission category and origin for NRTIs, subtype and CD4 count for NNRTIs, African origin for PIs, and HBV coinfection for INSTI SDRMs. Clinically meaningful resistance to raltegravir also differed according to age, educational level, CD4 count, and HBV coinfection status.

**Table 3.** Demographic, Clinical, and Virological Characteristics of ART-Naïve People With HIV (PWH) Included in CoRIS (2022–2023) and Univariate Analysis With Transmitted Drug Resistance

		% TDR (Transmitted Drug Resistance)				% CMR (Clinically Meaningful Resistance)	
	PWHIV	TDR NNRTI	TDR NRTI	TDR PI	TDR INI	CMR NRTI	CMR INI
Year							
2022	480 (46.7%)	39 (8.1%)	16 (3.3%)	12 (2.5%)	0 (0.0%)	3 (0.6%)	5 (1.0%)
2023	548 (53.3%)	49 (8.9%)	31 (5.7%)	6 (1.1%)	1 (1.2%)	9 (1.6%)	6 (0.9%)
Gender							
Female	116 (11.3%)	6 (5.2%)	2 (1.7%)	1 (0.9%)	0 (0.0%)	1 (0.9%)	1 (0.9%)
Male	851 (82.8)	63 (7.4%)	27 (3.2%)	12 (1.4%)	1 (0.1%)	7 (0.8%)	10 (1.2%)
Unknown	61 (6.0%)	…	…	…	…	4 (6.6%)	…

Age (years)							*P* = .0019 (≤25; RAL)
≤25	137 (13.3%)	11 (8.0%)	6 (4.4%)	2 (1.5%)	0 (0.0%)	2 (1.5%)	6 (4.4%)
26–35	374 (36.4%)	27 (7.2%)	7 (1.9%)	5 (1.3%)	1 (0.3%)	3 (0.8%)	5 (1.3%)
36–45	246 (23.9%)	14 (5.7%)	8 (3.3%)	6 (2.4%)	0 (0.0%)	3 (1.2%)	0 (0.0%)
46–55	142 (13.8%)	13 (9.2%)	8 (5.6%)	0 (0.9%)	0 (0.0%)	0 (0.0%)	0 (0.0%)
>55	68 (6.6%)	8 (11.8%)	0 (0.0%)	1 (1.5%)	0 (0.0%)	0 (0.0%)	0 (0.0%)
Unknown	61 (5.9%)	…	…	…	…	4 (6.6%)	…

Origin			*P* = .004652 (Europe [not Spain])	*P* = .01326 (Africa)		*P* = .04132 (Latin/Central America)	
Africa	17 (1.7%)	1 (5.9%)	0 (0.0%)	2 (11.8%)	0 (0.0%)	0 (0.0%)	0 (0.0%)
Europe (not Spain)	30 (3.0%)	2 (6.7%)	5 (16.7%)	0 (0.0%)	0 (0.0%)	0 (0.0%)	0 (0.0%)
Latin/Central America	359 (35.0%)	32 (8.9%)	11 (3.1%)	6 (1.7%)	0 (0.0%)	5 (1.4%)	4 (1.1%)
Others	118 (11.5%)	12 (10.2%)	3 (2.5%)	1 (0.9%)	0 (0.0%)	6 (5.1%)	1 (0.9%)
Spain	421 (41.0%)	23 (5.5%)	10 (2.4%)	3 (0.7%)	1 (0.2%)	1 (0.2%)	5 (1.2%)
Unknown	83 (8.1%)	…	…	…	…	…	…

Educational level							*P* = .0248 (secondary)
Nonstudies	9 (0.9%)	0 (0.0%)	1 (11.1%)	0 (0.0%)	0 (0.0%)	0 (0.0%)	0 (0.0%)
Primary	51 (5.0%)	5 (9.8%)	1 (2.0%)	2 (3.9%)	0 (0.0%)	0 (0.0%)	0 (0.0%)
Secondary	403 (39.2%)	26 (6.5%)	7 (1.7%)	5 (1.2%)	1 (0.3%)	1 (0.3%)	7 (1.7%)
University	244 (23.7%)	22 (9.4%)	14 (5.7%)	4 (1.6%)	0 (0.0%)	3 (1.2%)	1 (0.4%)
Unknown	321 (31.2%)	…	…	…	…	7 (2.2%)	2 (0.6%)

Transmission route			*P* value = .0264 (MSM)				
MSM	650 (63.2%)	50 (7.7%)	22 (3.4%)	9 (1.4%)	0 (0.0%)	7 (1.0%)	8 (1.2%)
MSW	208 (20.2%)	13 (6.2%)	3 (1.4%)	2 (1.0%)	1 (0.5%)	0 (0.0%)	2 (0.9%)
PWID	7 (0.7%)	1 (14.3%)	0 (0.0%)	0 (0.0%)	0 (0.0%)	0 (0.0%)	0 (0.0%)
Unknown	128 (12.5)	…	…	…	…	5 (3.9%)	…

CD4 counts (cell/mm^3^)						*P* = .0034 (200–350)	*P* = .0335 (<200)
<200	208 (20.2%)	18 (8.7%)	9 (4.3%)	7 (3.4%)	0 (0.0%)	4 (1.9%)	5 (2.4%)
200–350	250 (24.3%)	18 (6.0%)	9 (3.6%)	4 (1.6%)	0 (0.0%)	1 (0.4%)	3 (1.2%)
351–1000	458 (44.6%)	33 (7.2%)	10 (2.2%)	4 (0.9%)	1 (0.2%)	3 (0.7%)	2 (0.4%)
>1000	20 (1.9%)	2 (10.0%)	0 (0.0%)	0 (0.0%)	0 (0.0%)	0 (0.0%)	0 (0.0%)
Unknown	92 (8.9%)	…	…	…	…	…	…

Viral load (copies/mL)							
<100 000	450 (43.8%)	31 (6.9%)	17 (3.8%)	4 (0.9%)	1 (0.2%)	7 (1.6%)	6 (1.3%)
100 000–500 000	285 (27.7%)	23 (8.1%)	7 (2.5%)	7 (2.5%)	0 (0.0%)	0 (0.0%)	1 (0.4%)
>500 000	180 (17.5%)	15 (8.3%)	5 (2.8%)	2 (1.1%)	0 (0.0%)	0 (0.0%)	3 (1.7%)
Unknown	113 (11.0)	…	…	…	…	4 (2.7%)	…

Viral subtype		*P* = .03721 (subtype B)					
A1	19 (1.8%)	1 (5.3%)	0 (0.0%)	0 (0.0%)	0 (0.0%)	0 (0.0%)	0 (0.0%)
A6	17 (1.7%)	2 (11.8%)	0 (0.0%)	1 (5.9%)	0 (0.0%)	0 (0.0%)	0 (0.0%)
B	742 (72.2%)	64 (8.6%)	30 (4.0%)	10 (1.3%)	1 (0.1%)	8 (1.1%)	9 (1.2%)
C	33 (3.2%)	1 (3.0%)	0 (0.0%)	0 (0.0%)	0 (0.0%)	0 (0.0%)	0 (0.0%)
CRF02_AG	64 (6.3%)	1 (1.6%)	0 (0.0%)	2 (3.1%)	0 (0.0%)	0 (0.0%)	1 (1.6%)
F	15 (1.5%)	0 (0.0%)	0 (0.0%)	0 (0.0%)	0 (0.0%)	0 (0.0%)	0 (0.0%)
Others	69 (6.7%)	…	…	…	…	5 (7.2%)	…

HBV coinfection					*P* = 3.279e-05 (yes)		*P* value = 2.2e-16 (no)
NO	933 (90.8%)	72 (7.7%)	28 (3.0%)	13 (1.4%)	0 (0.0%)	7 (0.8%)	11 (1.2%)
SI	13 (1.3%)	0 (0.0%)	1 (7.7%)	0 (0.0%)	1 (7.7%)	1 (7.7%)	0 (0.0%)
Unknown	82 (8.0%)	…	…	…	…	…	…

HCV coinfection							
NO	929 (90.4%)	69 (7.4%)	29 (3.1%)	13 (1.4%)	0 (0.0%)	0 (0.0%)	0 (0.0%)
SI	24 (2.3%)	0 (0.0%)	0 (0.0%)	0 (0.0%)	1 (0.1%)	9 (1.0%)	10 (1.1%)
Unknown	75 (7.3%)	…	…	…	…	3 (4.0%)	…

Data are shown as *n*, %, and *P* value. The presence of transmitted drug resistance was assessed using surveillance drug resistance mutations (SDRMs) by drug class (NNRTI, NRTI, PI, and INSTI). CMR was defined as a Stanford HIVDB resistance score ≥ 3 and is shown for first-line NRTIs and INSTIs. Significant differences were determined using proportion comparison test. *P* values refer to comparisons across categories for each resistance outcome (χ^2^ or Fisher's exact test, as appropriate).

Abbreviations: CI, confidence interval; HBV, hepatitis B virus; HCV, hepatitis C virus; INSTI, integrase strand transfer inhibitor; MSM, men who have sex with men; NNRTI, non-nucleoside reverse transcriptase inhibitor; NRTI, nucleoside/nucleotide reverse transcriptase inhibitor; PI, protease inhibitor; PWID, person who injects drugs.

**Figure 1.**
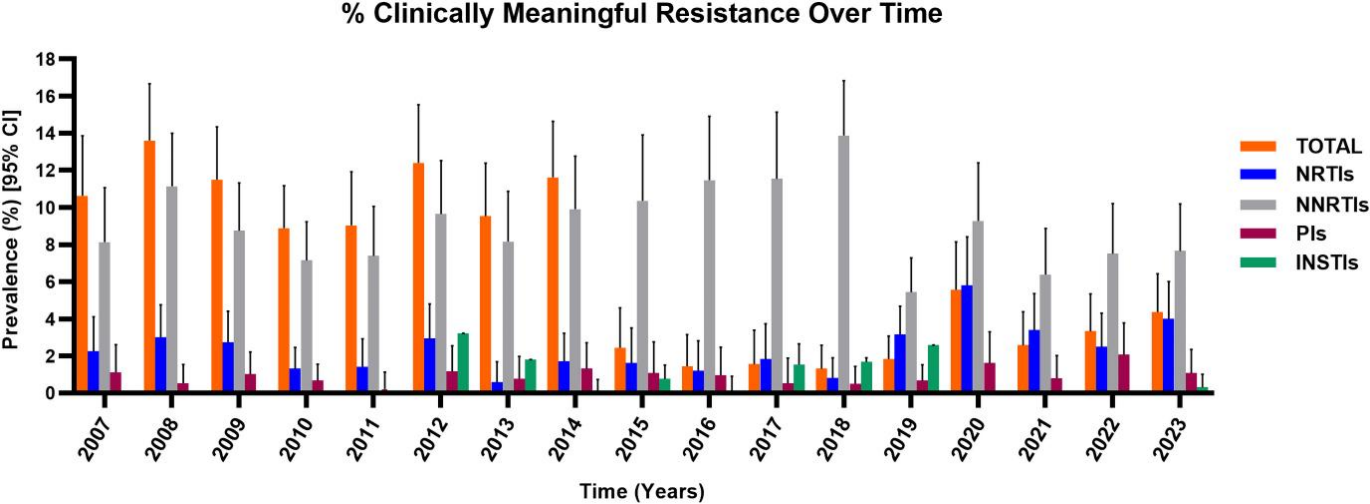
Grouped bar and line chart illustrating the prevalence of CMR from 2007 to 2023. The vertical axis (Y-axis) represents the prevalence percentage with 95% confidence intervals (95% CI). The chart highlights a positive public health trend, characterized by a sustained decline in overall CMR prevalence. While overall resistance reached peaks above 13% in the early years of the study (2007), it has consistently remained at lower levels in the most recent period, effectively stabilizing below 5% since 2015. The data for specific drug classes show that NNRTIs (gray) remain the most detected resistance type, though their impact on the overall trend has been mitigated by the decline in other classes. NRTIs (blue), PIs (maroon), and INSTIs (green); all show consistently low and stable prevalence levels throughout the last decade, contributing to the overall reduction in clinically meaningful resistance within the cohort.

The temporal trend of clinically meaningful TDR to first-line antiretrovirals in Spain (2007–2023) is shown in Figure 1. For each year, resistance was evaluated only for antiretroviral drugs recommended as first-line regimens according to the corresponding GeSIDA guidelines [5]. Throughout 2007–2023, clinically meaningful resistance to NNRTIs remained consistently high, whereas resistance to NRTIs remained relatively stable. In contrast, resistance to PIs and INSTIs declined progressively and became negligible after the adoption of boosted darunavir and second-generation INSTIs as first-line options [23]. Most notably, resistance to recommended first-line regimens as a whole declined from >12% in 2007 to virtually non-existent levels by 2023, coinciding with the absence of transmitted resistance to dolutegravir- or bictegravir-based regimens.

## DISCUSSION

In this nationwide analysis of ART-naïve PWH included in Spain, several key findings emerge. First, both the overall prevalence of TDR remained stable during the most recent study period (2022–2023), consistent with the temporal trends previously described in CoRIS analyses covering 2007–2021. Second, when examined over a longer timeframe, resistance to antiretroviral drugs recommended for first-line therapy declined markedly between 2007 and 2023, reflecting the progressive replacement of first-generation NNRTI-based regimens by integrase inhibitor–based strategies with higher genetic barriers to resistance. Third, rilpivirine-associated resistance mutations were relatively frequent, largely driven by the E138A substitution, highlighting the clinical relevance of NNRTI resistance beyond the classical SDRM framework. Finally, the distribution of M184V cases suggests that some resistance findings may need to be interpreted within evolving prevention strategies.

Our 2022–2023 TDR estimates align with those reported in other high-income countries, where transmitted resistance remains stable, is largely driven by NNRTI mutations, and rarely affects second-generation INSTIs [24–26]. In this context, persistent NNRTI-associated SDRMs should not be interpreted as evidence of major vulnerability of current first-line strategies, which are now predominantly INSTI-based, although they remain relevant for surveillance and selected NNRTI-containing regimens, particularly rilpivirine.

In Low- and Middle-Income Countries, where NNRTI-based regimens or historical NNRTI resistance may still have greater therapeutic relevance, the predominance of NNRTI resistance retains more direct clinical importance [27, 28].

Within this international context, our study provides additional clinically meaningful insights. An important aspect for interpreting our findings is that baseline INSTI resistance testing is currently performed in Spain more frequently than the restricted scenarios explicitly considered by most guidelines. In many participating centers, baseline resistance testing is now based on NGS platforms, and under these workflows, the marginal cost of sequencing integrase together with RT and protease is minimal. Therefore, the relatively high availability of baseline INSTI results in our cohort likely reflects routine laboratory practice rather than selective testing of patients considered at particularly high risk of harboring transmitted INSTI resistance. By extending resistance analyses beyond classical SDRMs to include rilpivirine-associated RAMs, we address an increasingly important gap in resistance surveillance in the era of long-acting cabotegravir/rilpivirine therapy. The relatively high prevalence of rilpivirine-associated RAMs, largely driven by E138A, underscores the need for careful resistance assessment when considering rilpivirine-containing regimens, particularly in the context of long-acting formulations. This issue has direct implications for candidate selection for long-acting cabotegravir/rilpivirine, as pooled analyses of phase 3 studies have shown that preexisting rilpivirine RAMs, HIV-1 subtype A6/A1, and body mass index ≥ 30 kg/m^2^ were baseline factors associated with an increased risk of confirmed virological failure, particularly when at least 2 of these factors coexisted [29]. In this context, our findings support systematic review of available baseline or historical genotypic resistance data for rilpivirine-associated RAMs before initiating long-acting cabotegravir/rilpivirine. Although our data do not by themselves justify universal de novo resistance testing solely for this purpose, they do reinforce the clinical value of prior resistance information when selecting candidates for long-acting therapy. We did not perform a formal subtype-specific analysis of rilpivirine-associated RAMs in the present study; however, it is noteworthy that subtype A1 and A6 together represented 3.5% of our cohort (1.8% and 1.7%, respectively), a non-negligible proportion given the previously reported association between A6/A1 subtype and virological failure risk in long-acting cabotegravir/rilpivirine. In parallel, the observed concentration of M184V cases among individuals with current or prior oral PrEP exposure suggests that some NRTI resistance findings may need to be interpreted within contemporary prevention frameworks, rather than being attributed solely to traditional transmission dynamics [15, 30]. However, because the absolute number of M184V cases was small and PrEP exposure was not systematically assessed in the full cohort, this observation should be considered hypothesis-generating and warrants confirmation in studies specifically designed to evaluate this question. Together, these findings illustrate the increasingly complex interface between HIV-1 prevention and treatment and the need for integrated surveillance approaches.

A major strength of this analysis is the incorporation of clinically meaningful transmitted resistance, defined using standardized interpretation algorithms. This concept has been systematically integrated into successive CoRIS resistance updates [19, 20] and more recently adopted within the MeditRes collaborative framework [24]. Unlike traditional SDRM-based surveillance, this approach directly assesses the functional impact of resistance mutations on currently approved first-line regimens, including both triple- and dual-drug combinations. Clinically, this distinction shows that while SDRM-defined transmitted resistance remains stable, clinically meaningful resistance affecting modern first-line regimens, especially integrase inhibitor–based therapies, has declined to very low levels. This observation reinforces the robustness of current treatment guidelines and supports the safety of modern INSTI-based regimens in treatment-naïve individuals. These results are especially relevant in the context of Test & Treat initiatives, where ART is initiated rapidly after diagnosis, often before baseline resistance results are available.

While concerns persist regarding the potential impact of transmitted NRTI resistance on 2-drug strategies, our analysis shows that M184V-associated clinically meaningful resistance is exceedingly rare among newly diagnosed individuals. Although several M184V cases occurred in persons with current or prior oral PrEP exposure, this finding should be interpreted cautiously given the limited number of cases and the absence of systematic PrEP exposure data for the full cohort. These data support systematic documentation of PrEP history at diagnosis, particularly when considering lamivudine-based dual therapy.

The long-term analysis spanning 2007–2023 further highlights the value of clinically meaningful resistance frameworks in Spain. Over this period, resistance to recommended first-line regimens declined significantly, largely driven by resistance to first-generation NNRTI-based regimens. This favorable evolution closely parallels the progressive replacement of low-genetic barrier regimens by boosted protease inhibitors and, subsequently, by second-generation INSTIs with high resistance barriers and excellent virological robustness [31, 32]. The sustained decline observed across nearly 2 decades of follow-up provides compelling population-level evidence that contemporary antiretroviral strategies have effectively mitigated the clinical consequences of transmitted resistance, strongly supporting the widespread implementation of Test & Treat strategies. Importantly, the demographic and clinical characteristics of our cohort were broadly representative of the population with newly diagnosed HIV-1 in Spain. In particular, age distribution, sex, and baseline viral load were consistent with recent national surveillance data, and these profiles were also comparable to those observed in previous years within CoRIS. This continuity supports the interpretation that the resistance patterns identified are unlikely to be explained by major shifts in the demographic or clinical composition of the study population [33].

The observed differences in the distribution of SDRMs and clinically meaningful transmitted resistance across demographic, clinical, and virological factors are consistent with prior CoRIS analyses and other European cohorts. NRTI and NNRTI patterns were broadly compatible with previous descriptions of transmission dynamics [34], whereas findings for PIs and INSTIs should be interpreted cautiously given the very low number of events.

Several limitations of this study should be acknowledged. CoRIS is an open, multicenter cohort, and disruptions to routine clinical activity during the COVID-19 pandemic affected sequence availability in some periods. Resistance data were derived from routine clinical practice, introducing heterogeneity in sampling and testing strategies, although CoRIS has been shown to be representative of the Spanish HIV-1 epidemic. In addition, baseline integrase sequences were not available for all participants, and coverage increased over time as broader NGS-based resistance testing was progressively implemented across centers. Therefore, estimates of transmitted INSTI resistance should be interpreted with some caution, particularly with respect to precision; however, the very low frequency of detected INSTI resistance suggests that the main clinical interpretation of our findings is unlikely to be substantially affected. Also, detailed information on PrEP exposure was only available for individuals with M184V mutations and was not systematically collected for participants without this mutation, limiting population-level inference regarding PrEP-associated resistance. Likewise, the small absolute number of M184V cases precludes firm conclusions regarding the strength or direction of this association. Finally, given the very low number of resistance events in several categories, multivariable modeling was not considered statistically robust. Therefore, the results from our univariate analyses should be interpreted as exploratory, unadjusted comparisons rather than estimates of independent associations. Despite these limitations, the study has important strengths, including its nationwide scope, long-term follow-up, high-quality data, and the use of a clinically meaningful resistance framework that directly informs contemporary clinical practice.

In conclusion, transmitted HIV-1 drug resistance in Spain remains stable in recent years, while its clinical impact on currently recommended first-line regimens has declined to near-zero levels. These findings provide strong population-level support for Test & Treat strategies and the immediate initiation of modern ART, while emphasizing the need for targeted resistance assessment in the context of rilpivirine-containing regimens and for further research on the potential relationship between prior PrEP exposure and selected NRTI resistance patterns. Although NNRTI resistance remains the main contributor to transmitted resistance, its present-day clinical significance is substantially greater in settings where NNRTI-based regimens still play a major role in first-line therapy.

## Notes

***Clinical implications***

- Clinically meaningful transmitted resistance to modern first-line antiretroviral regimens is now exceedingly rare, supporting immediate treatment initiation under Test & Treat strategies.
- Integrase inhibitor–based regimens, including both triple- and dual-drug combinations, remain highly robust against transmitted resistance in routine clinical practice.
- Rilpivirine-associated resistance mutations should be specifically assessed when considering rilpivirine-containing regimens, particularly long-acting cabotegravir/rilpivirine.
- Detection of M184V in newly diagnosed individuals should prompt careful assessment of prior oral PrEP exposure.

***Acknowledgments***

**CoRIS Executive Committee:**

Santiago Moreno, Inma Jarrín, David Dalmau, M Luisa Navarro, M Isabel González, Federico Garcia, Eva Poveda, Jose Antonio Iribarren, Félix Gutiérrez, Rafael Rubio, Francesc Vidal, Juan Berenguer, Juan González, M Ángeles Muñoz-Fernández.

**CoRIS Coordination Unit:**

Inmaculada Jarrín, Cristina Moreno, Marta Rava, Rebeca Izquierdo, Cristina Marco, Teresa Gómez-García. BioBanK HIV Hospital General Universitario Gregorio Marañón: M Ángeles Muñoz-Fernández, Roxana Juárez.

**Hospital General Universitario Dr. Balmis (Alicante):**

Joaquín Portilla, Irene Portilla, Esperanza Merino, Gema García, Iván Agea, José Sánchez-Payá, Juan Carlos Rodríguez, Livia Giner, Sergio Reus, Vicente Boix, Diego Torrus, Verónica Pérez, Julia Portilla, Héctor Pinargote.

**Hospital Universitario de Canarias (San Cristóbal de la Laguna):**

María Remedios Alemán, Ana López Lirola, Dácil García, Felicitas Díaz-Flores, M Mar Alonso, Ricardo Pelazas, María Inmaculada Hernández, Lucia Romero, Abraham Bethencourt, Daniel Rodríguez.

**Hospital Universitario Central de Asturias (Oviedo):**

Víctor Asensi, Rebeca Cabo Magadan, Lorena Fernández, Javier Díaz-Arias.

**Hospital Universitario 12 de Octubre (Madrid):**

Federico Pulido, Rafael Rubio, Otilia Bisbal, M Asunción Hernando, David Rial, María de Lagarde, Adriana Pinto, Laura Bermejo, Mireia Santacreu, Roser Navarro, Juan Martín Torres.

**Servicio de Enfermedades Infecciosas, Hospital Universitario Donostia, Instituto de Investigación BioDonostia (Donostia-San Sebastián):**

José Antonio Iribarren, M José Aramburu, Xabier Camino, Miguel Ángel Goenaga, M Jesús Bustinduy, Harkaitz Azkune, Maialen Ibarguren, Xabier Kortajarena, Ignacio Álvarez-Rodriguez, Leire Gil, Francisco Carmona-Torre, Ana Bayona Carlos, Maialen Lekuona Sanz, Claudia Nevado Pavón.

**Hospital General Universitario de Elche (Elche):**

Félix Gutiérrez, Catalina Robledano, Mar Masiá, Sergio Padilla, Rafael Pascual, Marta Fernández, Antonio Galiana, José Alberto García, Xavier Barber, Javier García Abellán, Guillermo Telenti, Ángela Botella, Paula Mascarell, Mar Carvajal, Lidia García-Sánchez, Nuria Ena, Leandro López, Jennifer Vallejo, Nieves Gonzalo-Jiménez, Montserrat Ruiz, Christian Ledesma, Santiago López, María Espinosa, Ana Quiles, María Andreo, María del Mar Alcalde, José García, Rosario Hernández, José Carlos Escribano, Marouane Menchi, María del Mar García Navarro.

**Hospital Universitari Germans Trias i Pujol (Can Ruti) (Badalona):**

Roberto Muga, Arantza Sanvisens, Daniel Fuster.

**Hospital General Universitario Gregorio Marañón (Madrid):**

Juan Carlos López Bernaldo de Quirós, Isabel Gutiérrez, Juan Berenguer, Margarita Ramírez, Paloma Gijón, Teresa Aldamiz-Echevarría, Francisco Tejerina, Cristina Diez, Leire Pérez, Chiara Fanciulli, Saray Corral.

**Hospital Universitari de Tarragona Joan XXIII (Tarragona):**

Joaquín Peraire, Anna Rull, Anna Martí, Consuelo Viladés, Beatriz Villar, Lluïsa Guillem, Silvia Chafino, Marina Flores.

**Hospital Universitario y Politécnico de La Fe (Valencia):**

Marta Montero-Alonso, María Tasias, Eva Calabuig, Miguel Salavert, Juan Fernández, Rosa Blanes, Jennifer Sánchez.

**Hospital Universitario La Paz/IdiPAZ (Madrid):**

Juan González-García, Ana Delgado-Hierro, José Ramón Arribas, Víctor Arribas, José Ignacio Bernardino, Carmen Busca, Joanna Cano-Smith, Julen Cadiñanos, Juan Miguel Castro, Luis Escosa, Iker Falces, Pedro Herranz, Víctor Hontañón, Alicia González-Baeza, M Luz Martín-Carbonero, Mario Mayoral, Rafael Micán, Rosa de Miguel, Rocío Montejano, Mª Luisa Montes, Luis Ramos-Ruperto, Berta Rodés, Talía Sainz, Elena Sendagorta, Eulalia Valencia, M del Mar Arcos, Alejandro de Gea Grela, Carlos Oñoro López.

**Hospital San Pedro Centro de Investigación Biomédica de La Rioja (CIBIR) (Logroño):**

José Ramón Blanco, Laura Pérez-Martínez, Valvanera Ibarra, Luis Metola, Mercedes Sanz, Estrella Melús.

**Hospital Universitario Miguel Servet (Zaragoza):**

Rosa Martínez, Desiré Gil, Ruth Caballero, María Aranzazu Caudevilla, María Espuelas Monge.

**Hospital Universitari Mutua Terrassa (Terrassa):**

David Dalmau, Marina Martinez, Angels Jaén, Mireia Cairó, Javier Martinez-Lacasa, Roser Font, Laura Gisbert.

**Hospital Universitario de Navarra (Pamplona):**

María Rivero, María Gracia, Carlos Ibero, Estela Moreno, Jesús Repáraz, Fernando Baigorria, Ana Miqueleiz, Miguel Fernández Huerta.

**Parc Taulí Hospital Universitari (Sabadell):**

Gemma Navarro, Sonia Calzado Isbert, Marta Navarro Vilasaro, Juan Francisco Delgado.

**Hospital Universitario de La Princesa (Madrid):**

Ignacio de los Santos, Alejandro de los Santos, Lucio García-Fraile, Enrique Martín, Ildefonso Sánchez-Cerrillo, Ana Barrios, Azucena Bautista, Carmen Sáez, Marianela Ciudad, Ángela Gutiérrez, María Aguilera García, Violeta Sampériz Rubio.

**Hospital Universitario Ramón y Cajal (Madrid):**

Santiago Moreno, Santos del Campo, José Luis Casado, Fernando Dronda, Ana Moreno, M Jesús Pérez, Sergio Serrano-Villar, Mª Jesús Vivancos, Javier Martínez-Sanz, Alejandro Vallejo, Matilde Sánchez-Conde, José Antonio Pérez-Molina, José Manuel Hermida, Erick De La Torre Tarazona, Elena Moreno, Laura Martín Pedraza, Claudio Díaz García, Jorge Díaz, Alejandro García, Raquel Ron.

**Hospital General Universitario Reina Sofía (Murcia):**

Enrique Bernal, Antonia Alcaraz, Joaquín Bravo, Ángeles Muñoz, Cristina Tomás, Eva Oliver, Eva García, Román González, Elena Guijarro, Rodrigo Martínez, María Dolores Hernández.

**Hospital Universitario Clínico San Cecilio (Granada):**

Federico García, Clara Martínez, Leopoldo Muñoz Medina, Marta Álvarez, Natalia Chueca, David Vinuesa, Adolfo de Salazar, Ana Fuentes, Emilio Guirao, Andrés Ruiz-Sancho, Francisco Anguita, Naya Faro, Lucia Chaves, Marta Illescas, Paloma Muñoz, Lucía Pérez.

**Centro Sanitario Sandoval (Madrid):**

Jorge Del Romero, Montserrat Raposo, Teresa Puerta, Mar Vera, Juan Ballesteros, Begoña Baza, Eva Orviz, Manuel Sanchez Robledo, Laura Dans Villán, Ruben Linares Navarro. Ines Armenteros Yeguas.

**Hospital Clínico Universitario de Santiago (Santiago de Compostela):**

Antonio Antela, Elena Losada.

**Hospital Universitario Son Espases (Palma de Mallorca):**

Melchor Riera, María Peñaranda, M Angels Ribas, Antoni A. Campins, Mercedes Garcia-Gasalla, Francisco J Fanjul, Javier Murillas, Luisa Martin-Pena, Francisca Artigues, Sophia Pinecki.

**Hospital Universitario Virgen de la Victoria (Málaga):**

Jesús Santos, María LópezJódar, Cristina Gómez-Ayerbe, Isabel Viciana, Rosario Palacios.

**Hospital Universitario Virgen del Rocío (Sevilla):**

Luis Fernando López-Cortés, Nuria Espinosa, Cristina Roca, Silvia Llaves, Marta Herreros, César Sotomayor.

**Hospital Universitario de Bellvitge (Hospitalet de Llobregat):**

Juan Manuel Tiraboschi, Arkaitz Imaz, María Saumoy, Analuz Fernandez, Jaime Vega Costa, Daniel Medina Gamito.

**Hospital Universitario Valle de Hebrón (Barcelona):**

Adrián Curran, Vicenç Falcó, Jordi Navarro, Joaquin Burgos, Paula Suanzes, Jorge García, Vicente Descalzo, Patricia Álvarez, Bibiana Planas, Marta Sanchíz, Arnau Monforte.

**Hospital Costa del Sol (Marbella):**

Julián Olalla, Javier Pérez, Alfonso del Arco, Javier de la Torre, José Luis Prada.

**Hospital General Universitario Santa Lucía (Cartagena):**

Onofre Juan Martínez, Lorena Martinez, Francisco Jesús Vera, Josefina García, Begoña Alcaraz, Sergio Guillén Martínez, Patricia Carles García.

**Complejo Hospitalario Universitario a Coruña (CHUAC) (A Coruña):**

Álvaro Mena, Berta Pernas, Pilar Vázquez, Soledad López, Brais Castelo.

**Hospital Universitario Basurto (Bilbao):**

Sofía Ibarra, Guillermo García, Josu Mirena, Oscar Luis Ferrero, Josefina López, Mireia de la Peña, Miriam López, Iñigo López, Itxaso Lombide, Víctor Polo, Joana de Miguel, Beatriz Ruiz Estevez, Maite Ganchegui Aguirre, María Jesús Barberá Gracia, Mónica Vélez Tobarias, Isabel Izuzquiza.

**Hospital Universitario Virgen de la Arrixaca (El Palmar):**

Carlos Galera, Marian Fernández, Helena Albendin, Antonia Castillo, Asunción Iborra, Antonio Moreno, M Angustias Merlos, Almudena Ortuño.

**Hospital de la Marina Baixa (La Vila Joiosa):**

Concha Amador, Francisco Pasquau, Concepción Gil, José Tomás Algado.

**Hospital Universitario Infanta Sofía (San Sebastián de los Reyes):**

Inés Suarez-García, Eduardo Malmierca, Patricia González-Ruano, M Pilar Ruiz, José Francisco Pascual, Luz Balsalobre, Ángela Somodevilla.

**Hospital Universitario de Jaén (Jaén):**

María de la Villa López, Mohamed Omar, Carmen Herrero.

**Hospital Universitario San Agustín (Avilés):**

Miguel Alberto de Zárraga, Desirée Pérez, Carlos Tarrazo Tarrazo, Juan Valdes Becares.

**Hospital Clínico San Carlos (Madrid):**

Vicente Estrada, Noemí Cabello, María José Núñez, Juncal Pérez-Somarriba, Reynaldo Homen, Ana Muñoz, Julia Barrado, Maravillas Carralon, Nieves Sanz, Susana Olmedo.

**Hospital Universitario Fundación Jiménez Díaz (Madrid):**

Miguel Górgolas, Alfonso Cabello, Beatriz Álvarez, Laura Prieto, Aws Al-Hayani, Irene Carrillo, Marta López de las Heras.

**Hospital Universitario Príncipe de Asturias (Alcalá de Henares):**

José Sanz, Cristina Hernández, María Novella.

**Hospital Clínico Universitario de Valencia (Valencia):**

M José Galindo, Sandra Pérez Gómez, Ana Ferrer.

**Hospital Reina Sofía (Córdoba):**

Antonio Rivero Román, Antonio Rivero Juárez, Pedro López, Mario Frias, Ángela Camacho, Ignacio Pérez, Diana Corona, Javier Manuel Caballero, Laura Ruiz Torres, Ángela Carrasco, Marina Gallo Marín, María Casares, Lucía Rios Muñoz.

**Hospital Universitario Severo Ochoa (Leganés):**

Rafael Rodríguez-Rosado Martinez-Echevarría, Rafael Torres.

**Hospital Universitario Virgen de Valme (Sevilla):**

Juan Macías Sánchez, Pilar Rincón, Luis Miguel Real, Anáis Corma, Jésica Martín.

**Hospital Álvaro Cunqueiro (Vigo):**

Eva Poveda, Alexandre Pérez, Luis Morano, Celia Miralles, Antonio Ocampo, María Gallego, Jacobo Alonso, Inés Martínez, Aida López.

**Hospital Clínico Universitario de Valladolid (Valladolid):**

Carlos Dueñas, Marta de la Fuente López, Cristina Novoa, Pablo Telleria, Marina García de Vicuña, Sara Gómez García.

**Hospital del Mar (Barcelona):**

Roberto Güerri Fernández, Claudia Navarro Valls, Juan Du, Agustin Marcos Blanco, Itziar Arrieta Aldea, Esperanza Cañas-Ruano, Cecilia Canepa, Natalia García Giralt.

***Patient consent statement*.** Before entering CoRIS, ethics approval was obtained from each of the participating sites and a written informed consent obtained from every patient included in the study. In addition, for this specific study, ethics approval was obtained from Hospital Universitario San Cecilio's Ethics Committee (internal reference no. *0053-N-23*).

***Data availability statement*.** The data underlying this article will be shared on reasonable request to the corresponding authors.

***Financial support.*** This work was supported by the Research Project PI22/00882, Instituto de Salud Carlos III. CoRIS cohort is supported by Centro de Investigación Biomédica en Red de Enfermedades Infecciosas (CB21/13/00091), Instituto de Salud Carlos III, Ministerio de Ciencia e Innovación and Unión Europea – NextGenerationEU. P. M. B. is supported by Instituto de Salud Carlos III (grant number FI23/00036). A. d. S. is supported by Instituto de Salud Carlos III (grant number JR22/00055). M. I. L. is supported by Instituto de Salud Carlos III (grant number CM24/00045). A. F. L. is supported by Instituto de Salud Carlos III (grant number JR24/00052).
